# Correction: Rational design of a stapled JAZ9 peptide inhibiting protein–protein interaction of a plant transcription factor

**DOI:** 10.1039/d1cb90002a

**Published:** 2021-01-26

**Authors:** Kaho Suzuki, Yousuke Takaoka, Minoru Ueda

**Affiliations:** Graduate School of Science, Tohoku University 6-3, Aramaki-Aza-Aoba, Aoba-ku Sendai 980-8578 Japan ytakaoka@tohoku.ac.jp minoru.ueda.d2@tohoku.ac.jp; Graduate School of Life Science, Tohoku University 6-3, Aramaki-Aza-Aoba, Aoba-ku Sendai 980-8578 Japan

## Abstract

Correction for ‘Rational design of a stapled JAZ9 peptide inhibiting protein–protein interaction of a plant transcription factor’ by Kaho Suzuki *et al.*, *RSC Chem. Biol.*, 2021, DOI: 10.1039/d0cb00204f.

The authors regret that the email address for corresponding author Minoru Ueda was omitted from the original article. The correct contact details are as shown here.

The Royal Society of Chemistry apologises for these errors and any consequent inconvenience to authors and readers.

## Supplementary Material

